# LIMLE, a New Molecule Over-Expressed following Activation, Is Involved in the Stimulatory Properties of Dendritic Cells

**DOI:** 10.1371/journal.pone.0093894

**Published:** 2014-04-04

**Authors:** Laëtitia Le Texier, Justine Durand, Amélie Lavault, Philippe Hulin, Olivier Collin, Yvan Le Bras, Maria-Cristina Cuturi, Elise Chiffoleau

**Affiliations:** 1 INSERM, U1064, Nantes, France; 2 CHU Nantes, Institut de Transplantation et de Recherche en Transplantation, ITUN, Nantes, France; 3 Université de Nantes, Faculté de Médecine, Nantes, France; 4 Plateforme MicroPICell, SFR santé, Nantes, France; 5 Plateforme GenOuest, IRISA-INRIA, Campus de Beaulieu, Rennes, France; University of Bergen, Norway

## Abstract

Dendritic cells are sentinels of the immune system distributed **throughout the body**, that following danger signals will migrate to secondary lymphoid organs to induce effector T cell responses. We have identified, in a rodent model of graft rejection, a new molecule expressed by dendritic cells that we have named LIMLE (RGD1310371). To characterize this new molecule, we analyzed its regulation of expression and its function. We observed that LIMLE mRNAs were rapidly and strongly up regulated in dendritic cells following inflammatory stimulation. We demonstrated that LIMLE inhibition does not alter dendritic cell maturation or cytokine production following Toll-like-receptor stimulation. However, it reduces their ability to stimulate effector T cells in a mixed leukocyte reaction or T cell receptor transgenic system. Interestingly, we observed that LIMLE protein localized with actin at some areas under the plasma membrane. Moreover, LIMLE is highly expressed in testis, trachea, lung and ciliated cells and it has been shown that cilia formation bears similarities to formation of the immunological synapse which is required for the T cell activation by dendritic cells. Taken together, these data suggest a role for LIMLE in specialized structures of the cytoskeleton that are important for dynamic cellular events such as immune synapse formation. In the future, LIMLE may represent a new target to reduce the capacity of dendritic cells to stimulate T cells and to regulate an immune response.

## Introduction

Dendritic cells (DCs) are central actors of the immune response. Localized at the interface with the external environment, DCs are sensors of pathogen penetration into the organism and are involved in both innate and adaptative immune responses. DCs detect microorganism components via pattern recognition receptors (PRRs), which transduce danger signals and induce their activation and maturation [1]. Moreover, DCs are professional antigen-presenting cells (APCs), able to capture and process antigen to present antigenic peptide on MHC Class I and Class II to activate CD8^+^ and CD4^+^ T cells respectively [2]. The fine-tuning of the maturation state of DCs is important to maintain the balance between immunity and tolerance. Under steady-state conditions, DCs remain in an immature state and do not mount an immune response against circulating self-antigens in the periphery, which maintains a state of tolerance. By contrast, foreign antigens result in maturation of DCs that will then migrate and activate T cells. This balance is required as any disturbance due to infection or tissue injury may result in chronic inflammation or autoimmunity.

Maturation of DCs is accompanied by numerous phenotypic and morphologic changes that are correlated with a reorganization of microfilaments and microtubules and an altered expression of specialized actin- and tubulin-associated proteins. Indeed, reorganization of the cytoskeleton is needed to allow dissolution of adhesion structures such as podosomes and acquisition of migratory ability [3]. Shape remodeling of DCs is also important in allowing acquisition of an elongated shape, as well as extension and retraction of long dendrites to form dynamic clusters with CD4^+^ T cells for efficient immune synapse formation and T cell activation [2], [4], [5], [6].

Comprehension of the mechanisms involved in DC maturation represents a crucial aim of research, to be able to develop new strategies to manipulate the immune response. Immune properties of DCs arouse interest due to their involvement in various pathologies such as infectious diseases, cancer, autoimmunity and graft rejection. As part of a study to identify genes associated with allograft rejection or tolerance in transplantation, we identified a new molecule that we named “LIMLE” for “LPS-Induced MoLeculE” and that we found to be over-expressed in the graft and blood of rats developing chronic rejection. In this study, we attempted to characterize the immune regulation and the function of this new molecule.

## Materials and Methods

### Ethics statement

The prefecture of the Loire-Atlantique and the Agriculture and Fishing Ministry has reviewed and approved the current study for animal experimentations (No. 4491). All efforts were made to minimize animal suffering during the experiments. Human blood of healthy donors were obtained from the Etablissement Français du Sang (EFS, Nantes, France), which has informed the donors prior the use of their blood. All donors have signed an informed consent. Further approval by the ethical committee was not necessary. A signed convention was established between our institution (INSERM) and the Etablissement Français du Sang (EFS, Nantes, France) to have access to the blood from healthy donors for research purpose.

### Animals and transplantation

MHC fully mismatched LEW.1A (RT1a) and LEW.1W (RT1u) rats and C57Bl/6 mice were purchased from the Centre d'Elevage Janvier (Le Genest-Saint-Isle, France). Transgenic mice S/SOPF B6.OT-I were purchased from Charles River Laboratories (L'Arbresle, France). Rodents were maintained in an animal facility under standard conditions according to our institutional guidelines. Heterotopic syngenic LEW.1A to LEW.1A or allogeneic LEW.1W to LEW.1A heart transplantations were performed using the Ono and Lindsey technique [7]. Allografts in untreated recipients were acutely rejected within 7 days. Allograft tolerance was induced, as previously described, by a short-term treatment (20 days, 3 mg/kg/day) with an immune-suppressor, LF15-0195 (Fournier Laboratories), a deoxyspergualine analog [8]. Chronic allograft rejection was induced by two donor blood transfusions (DST) before transplantation as previously described [9]. Graft function was assessed by scoring pulsations through the abdominal wall and acute or chronic rejection was confirmed by heart beat cessation or the presence of vascular lesions (histology) respectively. The graft and blood were harvested at day 5 or 100 after transplantation. Peripheral blood mononuclear cells (PBMC) were extracted from whole blood using Ficoll (GE Healthcare).

### Cell purification, culture, and activation

-Rat splenic DCs, total T cells, CD4^+^CD25^−^ and CD4^+^CD25^+^ T cells or B cells from naive rats were purified by positive selection on a FACSAria flow cytometer using Sirpα-FITC (OX62) and MHC Class II-APC/Cy7 (Ox6); TCR-Alexa Fluor 647 (R7/3); CD4-PE/Cy7 (W3/25) and CD25-PE (Ox39) or CD45RA-FITC (OX33) staining respectively. DCs, T cells and B cells were cultured in complete RPMI (RPMI plus 10% endotoxin-free Fetal Bovine Serum (Perbio Science), 2 mM l-glutamine, 1 mM sodium pyruvate, 1 mM HEPES, 5×10^−5^ M 2-mercaptoethanol andpenicillin-streptomycin (100 U/ml; 100 μg/ml respectively) (all from Sigma-Aldrich) and stimulated for 12 hours with lipopolysaccharide (LPS) (1 μg/ml) (Sigma-Aldrich), plate-bound anti-CD3 (1 μg/ml) and anti-CD28 (1 μg/ml) antibodies (BD Biosciences) or CpG 2006 (5 μM) (InvivoGen) respectively.

-Rat alveolar macrophage lineage NR8383 [10] cells were cultured in Ham's F12 complete medium and stimulated for 6 hours with rat IFN-γ (50 U/ml) (Serotec).

-Rat endothelial cell (EC) line of LEW.1W origin was isolated as previously described [11], plated into 12-well plates (Nunc; Merck/Eurolab France) (1 million cells/well) in complete RPMI medium for 24 hours and then stimulated for 6 hours with rat IFN-γ (50 U/ml) (Serotec).

-Rat bone marrow derived DCs (BMDCs) were obtained as previously described [12]. Bone marrow cells were cultured for 10 days in complete RPMI medium supplemented with 0.4 ng/ml of rat IL-4 – transfected COS- conditioned medium, and 1.5 ng/mL of murine granulocyte-macrophage (GM) colony-stimulating factor (CSF)–transfected COS-conditioned medium (at 1 million cells/ml) in 6 ml in 6 well-plates (Nunc). The plasmids containing the rat IL-4 and murine GM-CSF (pCDSR GMCSF) cDNA sequences were from our laboratory [13] and DNAX (Palo Alto, CA) respectively. After 8 days of culture, cells were recovered and plated without growth factors. After 48 hours of subculture two major cell populations were obtained: adherent and non-adherent cells. We demonstrated that the non-adherent populations, which are the ‘classical’ DCs, were able to stimulate naive allogeneic T cells and could be induced to completely mature using various stimuli [12], [14]. Therefore, for our experiments, rat non-adherent BMDCs were collected on day 8, plated (2 million cells/ml) in 6 ml in 6 well-plates and stimulated with LPS (1 μg/ml) (Sigma-Aldrich), recombinant rat IFN-γ (50 U/ml) (Serotec), CpG 2006 (5 μM) (InvivoGen), poly (I:C) (25 μg/ml) (InvivoGen) or recombinant rat IL-10 (20 ng/ml) (R&D Systems) for 6, 12, 24 and 48 hours.

-Human DCs were generated as previously described [15]. Briefly, monocytes were enriched by elutriation (>85% CD14^+^) and cultured for 6 days in RPMI complete medium supplemented with human GM-CSF (200 IU/ml) (CellGenix) and IL-4 (1000 IU/ml) (CellGenix). DCs were then harvested and stimulated at 1 million cells/ml for 24 hour with LPS (1 μg/ml). All cells were cultured at 37°C and 5% CO_2_, recovered and subjected to RNA extraction.

-For mouse BMDC generation, only murine GM-CSF (0.15 ng/ml) was added to complete RPMI medium during culture of bone marrow cells (at 1 million cells/ml) in 6 ml in 6 well-plates (Nunc).

### RNA extraction and real-time quantitative RT-PCR

Total RNA from organs, tissues or cells was prepared using TRIzol (Invitrogen) according to the manufacturer's instructions. Real-time quantitative RT-PCR was performed as previously described [16] by using a step one plus instrument (Applied Biosystems) and SYBR Green PCR Master mix reagent (Applied Biosystems). Oligonucleotides used in this study were for rat (r), mouse (m) and human (h): rHypoxanthine phosphoribosyltransferase (HPRT) (For-CCTTGGTCAAGCAGTACAGCC, Rev-TTCGCTGATGACACAAACATGA), rLIMLE (For-GTGTGACTGCTGTGCCGA, Rev-CCAGACAGAAGTCCTGCCC), mGlyceraldehyde 3-phosphate dehydrogenase (GAPDH) (For-CTACAGCAACAGGGTGGTGG, Rev-TATGGGGGTCTGGGATGG), mLIMLE (For-GCCCAGATGAAGTTAAAGCG, Rev-GCCCATAGACAACCACTTGG), hHPRT (For-CGAGATGTGATGAAGGAGATGG, Rev- CCTGTTGACTGGTCATTACAATAGC), hLIMLE (For-GCAGTTCAAGCCAAGACCC, Rev-AAATCCTCCTGTTATCCCCAG). HPRT (for rat and human) and GAPDH (for mice) were used as endogenous control genes to normalize for variations in the starting amount of RNA. Relative expression was calculated using the 2^−ΔΔ*C*t^ method [16], [17].

### Bioinformatics analysis

Rat, mouse and human LIMLE amino-acid (AA) sequences were analyzed with NCBI blast (National Center for Biotechnology Information database). To study cellular localization, the rat LIMLE AA sequence was analyzed using the AA sequence-based predictors PSORTII [18], TargetP1.1 [19] and NetNES [20]. The probability of LIMLE to be associated with the membrane was checked by the presence of alpha-helix with TMHMM 2.0 [21] and NMT tools [22]. The presence of specific signals or links with known family proteins according to the predictive secondary structure or predictive 3D structure, were analyzed by InterProScan portal [23], Gene3D [24], “Protein Homology/analogY Recognition Engine” (PHYRE) [21] [25], [26] and the “Iterative Threading ASSEmbly Refinement” (I-TASSER) [27], [28] web servers.

### Plasmid construction and transfection

Full-length rat LIMLE mRNA sequence was cloned from rat testis cDNA by PCR with the specific primers: rLIMLE (Up-ACGCGGATCCATGGCGCGGAATGTGTATGGTCC, Lo-ATAGTTTAGCGGCCGCCCTGTATTGATGCCCATGGCCTGCTC) (Eurofins MWG) and inserted into the pcDNA6 vector (Invitrogen). 6×10^4^ COS (CV-1 (simian) in origin, and carrying the SV40) fibroblast-like cell line derived from monkey kidney tissue (kindly provided by the Etablissement Français du Sang (EFS, Nantes, France) which acquired it from **L**ife Technologies)), or rat BMDC (day 8 of culture) were plated on Microscope Cover glasses (Marienfield GmbH & Co.KG) in 12-well plates (Nunc; Merck/Eurolab France) for 24 hours. Cells were transfected with pcDNA6 plasmid with lipofectamin (Invitrogen) and Plus reagent (Invitrogen) for COS and with Nucleofector Kit Dendritic cell (Lonza) for BMDCs, according to the manufacturer's instructions. Cells were stained 24 hours after transfection.

### Immunohistology

COS cells or rat BMDCs were fixed with paraformaldehyde (4%) (Electron Microscopy Science, Hatfield, USA), permeabilized with Triton (0.3%) and stained with anti-V5 antibody (11 μg/ml) (Invitrogen), Alexa Fluor 568 anti-mouse IgG antibody (4 μg/ml) (Invitrogen) and FITC phalloidin (0.4 U/ml, Invitrogen). Cover glasses were mounted in Prolong Gold 4′-6′-diamidino-2-phenylindole (DAPI) mounting medium (Invitrogen), and observed by fluorescence microscopy (*Nikon* A1 R Si Confocal microscope). Images were obtained (X60 Plan Apo N.A: 1.4, zoom 2.) with sequential mode and analyzed by using Metamorph program.

### RNAi BMDC and COS transfection, activation and T cell stimulation

Non-overlapping LIMLE specific Stealth RNAi (Stealth Select RNAi; Invitrogen) duplexes were both designed and purchased by Invitrogen: Rat RNAi-1 (5′-CCACUGGAUGCUGGUUACUGGAAAU-3′), rat RNAi-2 (5′-CAUGCUAGACAUCUCUAAACCUAUU-3′), mouse RNAi-1 (5′-GCGAAAUGGGCCAAGUGGUUGUCUA-3′) and mouse (RNAi-2 5′-UAGACAACCACUUGGCCCAUUUCGC-3′). Two million COS or adherent rat or mouse BMDCs (at day 8 of culture) were transfected with Lipofectamine RNAiMAX (Invitrogen) and 200 pmol of control RNAi (medium GC content Stealth RNAi negative universal control, Invitrogen) or LIMLE specific RNAi. BMDCs were stimulated for 48 hours with LPS (0.5 μg/ml). Two days following RNA transfection, COS cells were fixed with paraformaldehyde (4%), scraped, permeabilized with saponin (0.5%) and stained with anti-V5 antibody (11 μg/ml) (Invitrogen) and Alexa Fluor 568 anti-mouse IgG antibody (4 μg/ml) (Invitrogen) for flow cytometric analysis.

-For rat mixed leukocyte reaction (MLR), LEW.1A BMDCs were harvested 2 days after transfection and plated in 96-well plates (6.25×10^4^ cells/ml) with allogeneic LEW.1W lymph node-purified total, CD4^+^CD25^−^ or CD4^+^CD25^+^ T cells (20×10^4^ cells/ml) in complete RPMI medium. For the MLR with total T cells, cells were pulsed three days later for 8 h with 0.5 μCi/well [methyl-^3^H]-thymidine (Amersham) and ^3^H incorporation was measured using a scintillation counter (TopCount NXT; PerkinElmer). INFγ, IL-6 and IL-12 production was assessed in the MLR supernatants (at day 5) by ELISA according to the manufacturer instructions (BD Biosciences). For MLR with Carboxyfluorescein succinimidyl ester (CFSE)-labeled (Invitrogen) CD4^+^CD25^−^ or CD4^+^CD25^+^ T cells, cells were stained on day 4 of culture, with R73-biotin and streptavidin/Alexa Fluor 405 (TCRαβ^+^) and W3/25-PE/Cy7 (CD4^+^) and T cell proliferation (CFSE dilution) was analyzed by flow cytometry.

-In the mouse experiments, efficiency of mouse BMDC RNAi transfection was determining by transfecting cells with an Alexa fluor Red control Oligo (Invitrogen). Analysis was performed 48 hours later by FACS.

-In vitro antigen-specific T cell stimulation assay: BMDC, 8-days post-transfection, were loaded with OVA_257–264_ peptide (0.1 ng/ml) or ovalbumin protein (500 μg/ml), stimulated with LPS (0.5 μg/ml) for 24 hours, washed and then incubated (1×10^4^ cells) with CFSE-labeled lymph node purified OT-I CD8^+^ T cells (10×10^4^ cells) (Miltenyi kit) in 96-well plates. On day 4 of culture, cells were stained with anti-CD3-APC (BD Biosciences) and anti-CD8-PE/Cy7 (BD Biosciences) and T cell proliferation (by CFSE dilution) was analyzed by flow cytometry.

### Flow cytometry analysis

Fluorescent labeling was measured using a FACS LSR II (BD Biosciences) and analyzed with FlowJo Software.

### Statistical analysis

Statistical evaluation was performed using Student's *t* test for unpaired data, and results were considered significant if *p* values were <0.05. Data are expressed as mean ± SEM.

### Online Supplemental Material

All the material and methods used for the supplemental data are described in the Material and Methods section.

## Results

### Identification of an uncharacterized molecule over-expressed in graft rejection

**Figure 1:**
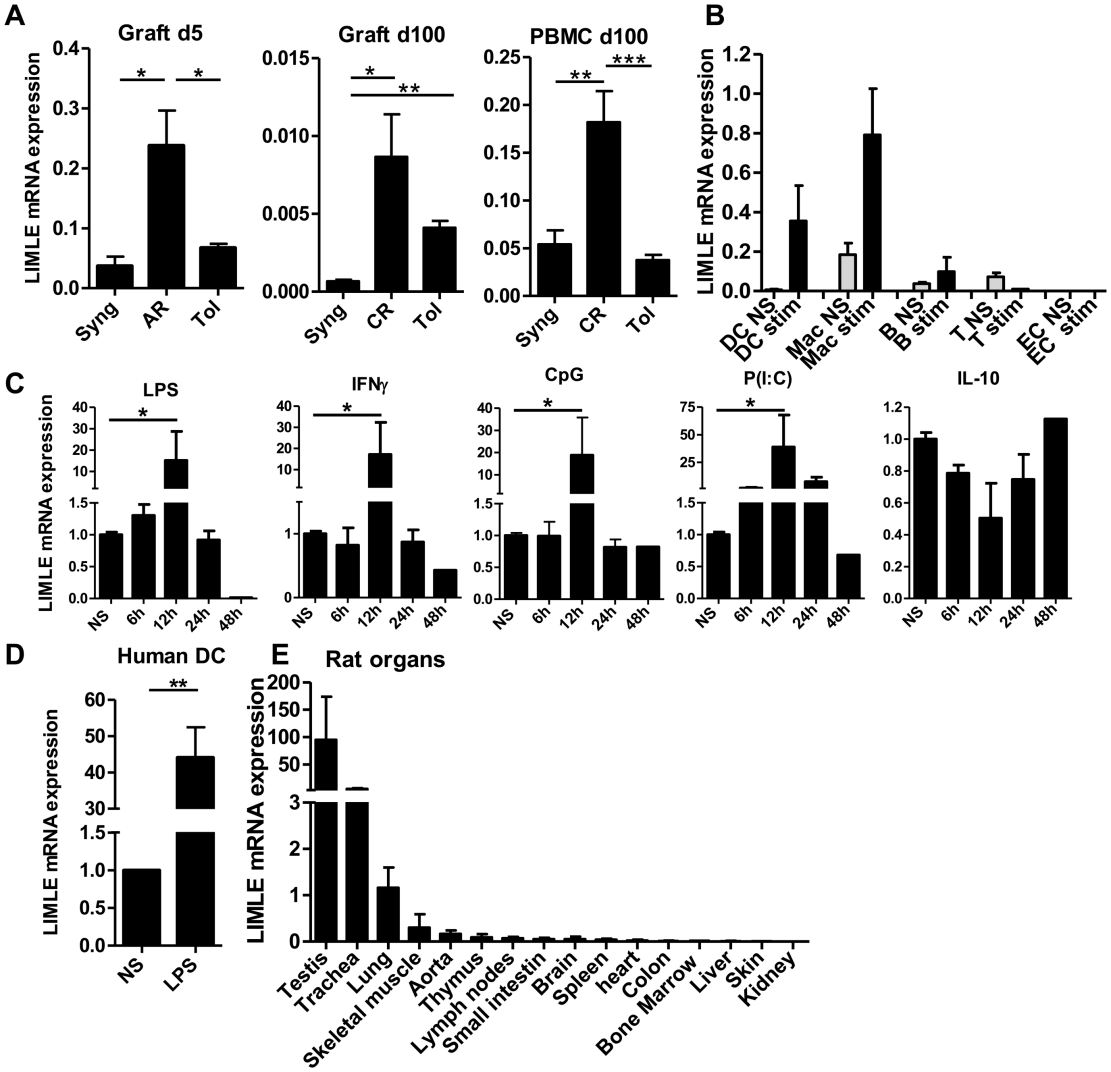
LIMLE mRNA expression in transplantation models, cell subtypes and organs. LIMLE mRNA expression in (A) cardiac grafts and PBMC of rat syngenic recipients (Syng), allograft recipients developing acute (AR) or chronic rejection (CR), or allograft tolerant recipients (Tol) at day 5 or 100 after transplantation (n = 5, *p<0.05 **p<0.01 ***p<0.001), (B) Splenic DCs, alveolar macrophage lineage cells NR8383 (Mac), B cells, T cells and EC, stimulated (stim) or not (NS) with LPS, IFNγ, CpG, anti-CD3/CD28 or IFNγ respectively (for 12 hours) (n = 3), (C) rat BMDCs stimulated or not (NS) with LPS, IFNγ, CpG, Poly(I:C) or IL-10 for 6, 12, 24 or 48 hours (n = 3, *p<0.05), (D) Human DCs stimulated or not (NS) with LPS for 12 hours (n = 3, **p<0.01) (E) mRNA expression of LIMLE in different organs from naive rats as indicated (n = 5). (A–E) Quantitative RT-PCR results were expressed in Arbitrary Units (AU) of LIMLE/HPRT transcript ratio ± SEM. Statistical evaluation was performed using Student's *t* test for unpaired data, and results were considered significant if *p* values were <0.05.

In an attempt to identify new genes that could be involved in the development of chronic rejection or tolerance in transplantation, we applied a pan genomic DNA chip. In a rat MHC fully mismatched cardiac allograft model, we compared whole allografts that develop chronic rejection with tolerated allografts induced by a short-term treatment with an immunosuppressor [9]. We have previously extensively described these models and shown that the DNA chip enabled the identification of molecules involved in these complex phenomena [9], [29]. Among the genes up-regulated in chronically rejected allografts versus tolerated ones, we identified the rat gene ID 308794 (RGD1310371). This gene was previously identified as being highly expressed by mouse ciliated cells but has not been characterized [30]. We arbitrarily named this molecule “LIMLE” for “LPS-Induced MoLeculE”. We confirmed, by quantitative RT/PCR, the over-expression of LIMLE mRNA in chronically rejected allografts compared to syngenic grafts (Fig. 1A, n = 5, *p<0.05). We also observed a peak of expression in acutely rejected allografts (Fig. 1A, n = 5, **p<0.01). Interestingly, we also observed a higher mRNA expression of LIMLE in the blood of recipients developing chronic rejection compared to syngeneic or tolerant recipients (Fig. 1A, n = 5, **p<0.01 and ***p<0.001). These results suggest on over-expression of LIMLE during an inflammatory immune response.

### LIMLE is highly expressed in activated myeloid cells and in testis, trachea and lung

In order to characterize this new molecule, we analyzed its expression in different rat cell types. Quantification of LIMLE mRNA showed a very low expression in all tested resting cells (NS) (spleen DCs, alveolar macrophage lineage Mac (NR83 83), B, T and EC) (Fig. 1B). However, we observed that LIMLE mRNA expression was strongly induced (up to 50 fold) following inflammatory stimulation (Stim) in DCs and alveolar macrophage lineage cells but not in B, T or ECs (Fig. 1B). We performed a LIMLE mRNA expression kinetic analysis in BMDCs and observed that LIMLE was strongly induced (up to 100 fold) 12 hours after stimulation with the different pro-inflammatory stimuli tested (LPS, IFNγ, CpG or Poly(I:C)) (n = 3, *p<0.05), following which expression rapidly decreased. Interestingly, in contrast to pro-inflammatory stimuli, LIMLE mRNA expression tended to decrease following 12 hours of stimulation with the immuno-regulatory cytokine IL-10 (Fig. 1C) (n = 3, not significant). We analyzed LIMLE mRNA expression in human monocyte-derived DCs and also observed a strong up-regulation (up to 45 fold) following LPS-stimulation, suggesting similar regulation of LIMLE expression across species (Fig. 1D, n = 3, **p<0.01). We then analyzed LIMLE mRNA expression in different organs or tissues from naive rats. We noted strong expression in the testis, trachea and lung. The expression was lower in the other organs or tissues tested (skeletal muscle, aorta, thymus, lymph nodes, small intestine, brain, spleen, heart, colon, bone marrow, liver, skin, and kidney) (Fig. 1E, n = 3).

### LIMLE is involved in the stimulatory properties of DCs

**Figure 2:**
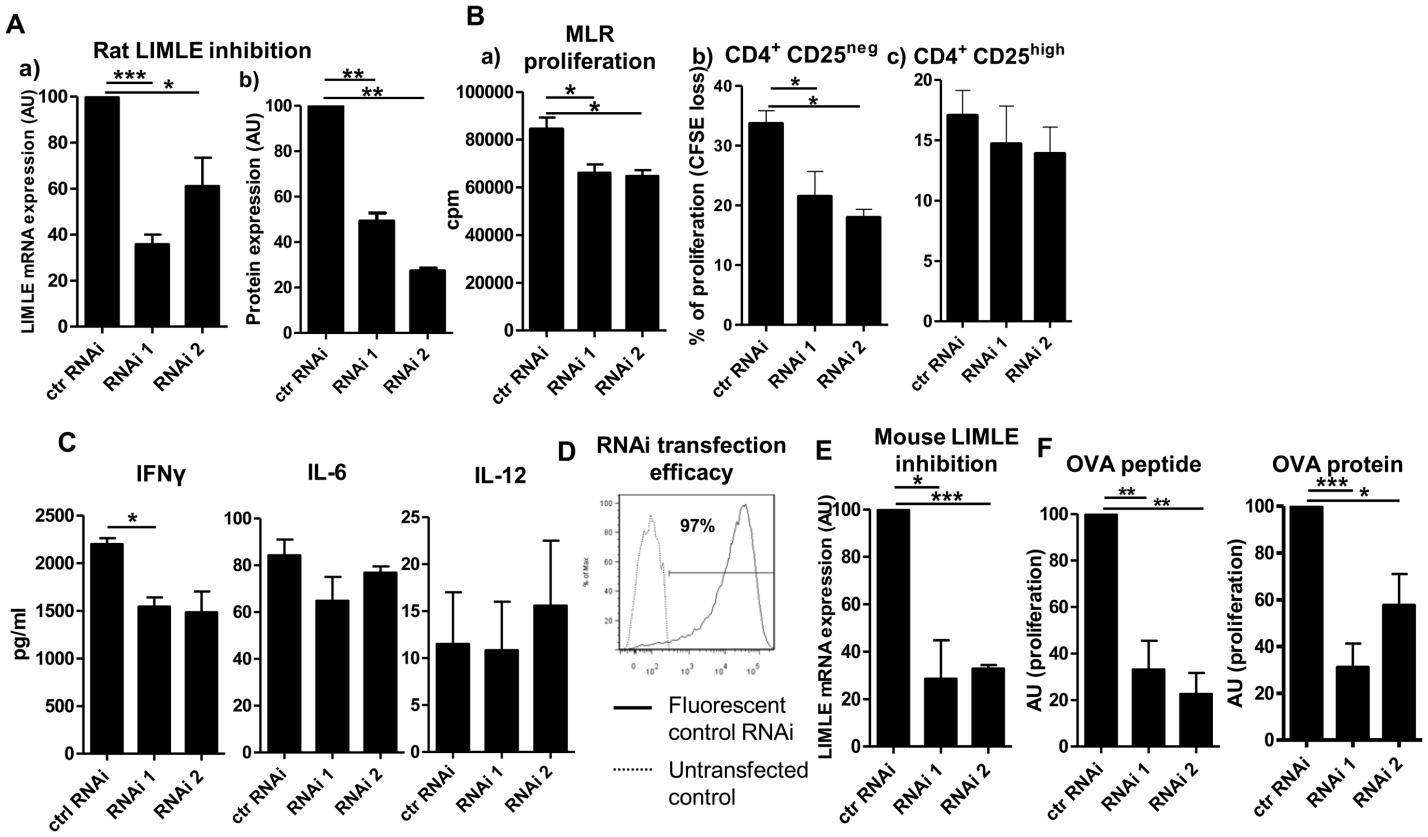
The role of LIMLE in the stimulatory properties of BMDCs. (A) a) LIMLE mRNA expression in rat BMDCs transfected with control or one of two LIMLE specific RNAi. Quantitative RT-PCR results were expressed in Arbitrary Unit (AU) of LIMLE/HPRT transcript ratio ± SEM (n = 3 *p<0.05 ***p<0.001); b) V5 tagged LIMLE protein expression in eukaryote cells transfected with a plasmid encoding rat LIMLE and V5 and either a control or one of two LIMLE specific RNAi. Data were expressed in Arbitrary Units (AU) of % of V5^+^ cells assessed by flow cytometry compared to control RNAi  = 100 (n = 6, **p<0.01) (B) Proliferation of allogeneic T cells stimulated with control or one of two LIMLE specific RNAi transfected BMDCs (MLR) for 4 days as follow a) ^3^H incorporation (cpm) for bulk T cells, b) CFSE loss from CD4^+^CD25^neg^ and c) CFSE loss from CD4^+^CD25^high^ T cells (n≥3, *p<0.05). (C) IFNγ, IL-6 and IL-12 quantification by ELISA of MLR supernatants (n = 3, *p<0.05). (D) Representative histograms (flow cytometry) of mouse BMDC transfection efficiency (with red fluorescent control RNAi (plain line) or untransfected control (dotted line)). (E) LIMLE mRNA expression in mouse BMDCs transfected with control or one of two LIMLE specific RNAi. Quantitative RT-PCR results were expressed in Arbitrary Units (AU) of LIMLE/GAPDH transcript ratio ± SEM (n = 3, *p<0.05 ***p<0.001). (F) Proliferation of OT-I CD8^+^ T cells stimulated for 4 days with OVA_257–264_ peptide (0.1 ng/ml) (n = 3) or OVA protein (500 μg/ml) (n = 4) loaded mouse BMDCs (transfected with control or one of two LIMLE specific RNAi). Data were expressed in AU of CFSE dilution compared to control = 100 (*p<0.05 **p<0.01 ***p<0.001). Statistical evaluation was performed using the Student's *t* test for unpaired data, and results were considered significant if *p* values were <0.05.

To determine the function of LIMLE, we used specific LIMLE reformed-type small interfering RNA (RNAi) that allows efficient and long-term inhibition of the specific gene without inducing maturation of the cells [31]. Two non-overlapping RNAi specific for LIMLE were tested in rat LPS-stimulated BMDC and compared to a control RNAi. We previously demonstrated that this technology transfects most of the rat BMDCs (up to 80% of the cells were transfected with a fluorescent RNAi) and allows efficient mRNA and protein inhibition [29], [32]. With LIMLE specific RNAi, we obtained efficient inhibition of LIMLE mRNA expression in LPS-stimulated BMDCs compared to control RNAi (up to 70%) (Fig. 2A a), n = 3, *p<0.05 and ***p<0.001). To check for LIMLE protein inhibition, we attempted to generate anti-rat LIMLE polyclonal antibodies by immunizing rabbits with LIMLE specific peptides. Unfortunately, we did not obtain efficient immunization, suggesting that LIMLE is highly conserved between species. Therefore, we transfected eukaryote cells by lipofection with a plasmid encoding the rat LIMLE full-length sequence and containing the V5 tag. We then tested the inhibition efficiency of the LIMLE specific RNAi on LIMLE protein via the V5 tag. We obtained efficient inhibition of LIMLE protein expression compared to control RNAi (up to 70%) (Fig. 2A b), n = 6, **p<0.01). We then analyzed whether LIMLE inhibition modified LPS-induced maturation of immature BMDCs. To generate immature BMDCs, we used low concentrations of GM-CSF and IL-4 to obtain the “non-adherent” population of immature cells that are able to further maturate following LPS stimulation and to efficiently stimulate T cells. Although LIMLE mRNA expression is highest at 12 hours after stimulation, we incubated BMDC with RNAi for 48 hours to be sure that the mRNA expression and consequently the protein expression would be decreased. Moreover, we used 48 hours to be sure that the BMDC had the time to fully maturate with the Toll-like receptor ligand (TLR-L) stimulus (LPS) and hence be able to stimulate T cells. We did not observe any effect of LIMLE inhibition on viability of the cells, on the expression of activation molecules such as MHC Class I and Class II, CD80 or CD86, or on the LPS-induced cytokine production of IL-12p70, IL-6 or IL-10 (Fig. S1, A, B and C respectively). Next, we investigated whether inhibition of LIMLE in matured BMDCs affected their ability to promote T cell proliferation. In a fully allogeneic mixed leukocyte reaction (MLR), we observed a reduction in T cell proliferation when LIMLE was inhibited in the matured BMDCs (Fig. 2B a), n = 3, *p<0.05). Since we observed an increase in LIMLE expression with pro-inflammatory stimuli and a tendency for a decrease with IL-10, LIMLE could be involved in the expansion of effector T cells and not of regulatory T cells, to favor a strong immune response. To determine whether LIMLE inhibition decreased the ability of BMDCs to stimulate both allogeneic effector and regulatory T cells, we also stimulated highly purified CD4^+^CD25^−^ and CD4^+^CD25^high^ (mostly regulatory T cells) in a MLR. We observed that LIMLE inhibition in BMDCs decreased significantly the proliferation of purified CD4^+^CD25^−^ (Fig. 2B b), n = 4, *p<0.05). This decreased is not evident with purified CD4^+^CD25^high^ regulatory T cells (Fig. 2B c), n = 4). This could be due to their low proliferation. Nevertheless, increase of proliferation was not observed.

Low production of IFNγ was noted in the MLR supernatants due to the low proliferation of the allogeneic effector T cells (Fig. 2C, n = 3, *p<0.05). However, we observed again no modification of IL-6 and IL-12 expression by the matured BMDCs in the MLR. These results demonstrate that LIMLE is not involved in the maturation of the DCs but rather in their capacity to stimulate effector T cells (Fig. 2C, n = 3).

We then used a more relevant and sensitive system of T cell stimulation using CD8^+^ T cells from OT-I transgenic mice that are TCR transgenic for ovalbumin (OVA). First, we evaluated the efficiency of transfection of mouse BMDCs with specific fluorescent RNAi. We observed that almost all of the cells (up to 97%) were transduced with RNAi (Fig. 2D). We confirmed inhibition of LIMLE mRNA expression in mouse BMDCs (up to 70%) with two different mouse LIMLE specific RNAi (Fig. 2E, n = 3, *p<0.05 and ***p<0.001). Similar to our observation in rat BMDCs, inhibition of LIMLE in mice DCs did not modify the viability of the cells or the expression of activation molecules (MHC Class I and Class II, CD80 or CD86) (Fig. S2, A and B respectively). However, LIMLE inhibition in OVA_257–264_ peptide or OVA protein loaded BMDCs strongly reduced their ability to stimulate the transgenic CD8^+^ T cells (Fig. 2F, n = 3, *p<0.05, **p<0.01, ***p<0.001) (Fig. S3, gate strategy (A) and dose effect (B) respectively). The reduction of proliferation was observed with both OVA peptide or OVA protein loaded DC, demonstrating that this decrease was not due to a fault in antigen processing. These data confirm a role for LIMLE in DCs for the activation of effector T cells.

### LIMLE protein localization and bioinformatic analysis

To determine the cellular localization of LIMLE, we analyzed its amino-acid (AA) sequence by using a variety of predictive software programs including PSORTII [18], TMHMM2.0 [21], NMT tools [22], TargetP1.1 [19] and NetNES [20]. Results revealed no transmembrane domain and a high probability for LIMLE to be located in the cytoplasm (data not shown). In addition, we detected a Nuclear Export Signal domain (NES) from AA 100 to 110 (**I**-K-**A**-Q-**L**-N-D-D-**L**-E-**I**), suggesting that LIMLE can circulate between the nucleus and cytoplasm.

**Figure 3:**
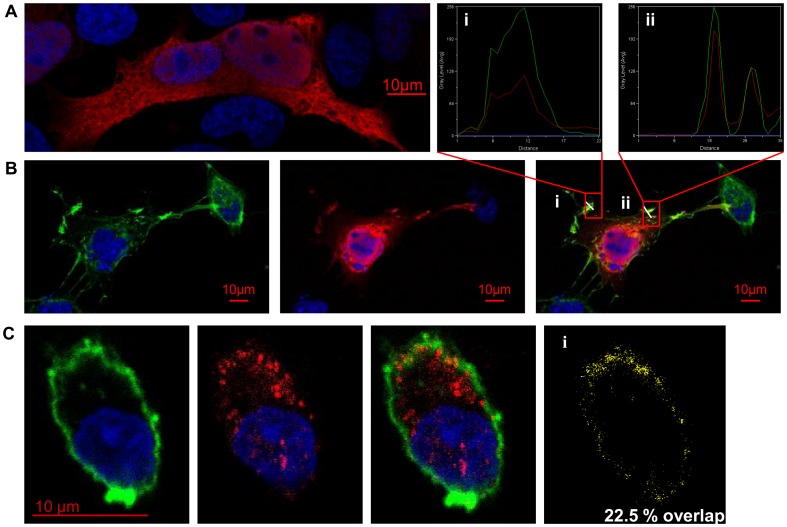
Cellular localization of LIMLE protein. Representative pictures of immuno-fluorescence staining of (A and B) COS or (C) BMDCs transfected with a plasmid encoding full length rat LIMLE protein and containing the V5 tag (red) with DAPI (blue) and and phalloidin (polymerized actin) (green). Original magnification ×1200 (Plan Apo N.A: 1.4 zoom 2.). (Bi,ii) Representative histograms of fluorescence intensity (AU) in COS cells detected in pixels with the indicated box, graphed with the linescan function of Metamorph Image Processing Software. (Ci) Representative picture of LIMLE overlapping with actin in DCs, processed with Metamorph Image Processing Software. Pictures were representative of three independent experiments.

To determine its exact cellular localization, without access to specific antibodies, we transfected eukaryote cells (COS) by lipofection with a plasmid encoding the rat LIMLE full-length sequence and containing the V5 tag. Confocal microscopy analysis revealed expression of the V5 tag-associated LIMLE protein in the cytoplasm and nucleus. Interestingly, tagged LIMLE protein appeared to be organized as a network in the cytoplasm of adherent COS cells, similarly to some cytoskeleton filament proteins (Fig 3A). Therefore, we performed staining of LIMLE protein and polymerized actin using phalloidin. Interestingly, we observed that LIMLE localized with actin at some areas under the cell plasma membrane (at specific cytoskeleton organized structures or protrusions) (Fig 3B). The profiles of the fluorescent intensities for LIMLE and actin were plotted at two different cell membrane locations (transept white lines) (Fig 3B i) ii)). Representative histograms showed overlapping staining and parallel fluctuations of LIMLE and actin intensities suggested a close relationship between these proteins at some areas under the plasma membranes. We attempted to confirm this staining in more physiologic eukaryote cells than the COS by using rat BMDCs. Rat BMDCs were transfected by nucleofection with the plasmid encoding rat LIMLE and confocal microscopy analysis revealed the same type of staining as in COS cells, with staining in the nucleus and cytoplasm (Figure 3C). We also observed some localization of LIMLE with actin at some areas under the plasma membrane in DCs (22.5% of LIMLE protein overlaps with actin in representative DC) (Fig 3C i)).

In the literature, nothing has been described about the LIMLE gene except its high expression by mouse ciliated cells [30]. To obtain more information about LIMLE and notably, to determine whether this new molecule could be linked to a specific family, we performed *in silico* analysis of the LIMLE sequence in collaboration with the GenOuest bioinformatics core facility (Rennes, France). NCBI blast of the rat LIMLE sequence (gene name RGD1310371, gene ID 308794) revealed 71% and 88% identity with the human (gene name C15orf26, gene ID 161502) and mouse (gene name 1700026D08Rik, gene ID 75556) sequences respectively, demonstrating that LIMLE is highly conserved between species. Any specific signal characteristic of a known family protein or specific pattern was revealed with InterProScan portal [23]. Moreover, sequence alignment algorithms such as “Basic Local Alignment Search Tool” (BLAST) [33] and PSI-BLAST [34] could not detect a homolog of LIMLE in the protein databank.

Sometimes, structural information can help to predict biological function of a molecule (3D shape dictates how the protein interacts with a ligand or other proteins). We used the homology modeling web servers “gene3D” [24], the I-TASSER [27], [28] and PHYRE for protein structure prediction [25], [26], [35]. Results from these 3 different web servers revealed a high probability for a structural link between LIMLE and the trefoil protein family (with 6 hits exhibiting a % of identity ≥ 20%, the threshold to consider homology). Trefoil molecules are characterized by a rich cysteine domain, named the trefoil domain, and by their “three-leaf” structure [36], [37], [38]. LIMLE does not contain a rich cysteine domain, so does not belong to the trefoil molecules. However, by its predictive structure, it presents similarities to the structure of the trefoil molecule family.

We also observed conserved gene order of the following genes Tmc3, Stard5, IL-16, LIMLE (RGD1310371), Mesdc1 (mesoderm development candidate 1) and Mesdc2 in rat, mouse and human chromosomes (Data not shown). Interestingly, the LIMLE gene is flanked by IL-16 and Mesdc-1, two genes that have been described to bind the cytoskeleton proteins myosin [39] and actin [40], [41] respectively. Exceptional conservation of shared synteny can reflect important functional relationships between genes [42]. Additionally, genes of similar structure or linked functions are often grouped in a locus/complex of genes or supergenes on a chromosome as is the case for the NK or MHC gene complexes [43], [44]. Therefore, the proximity of LIMLE with these genes may suggest the presence of a locus/complex of genes sharing similar properties or functions related to the cytoskeleton.

## Discussion

In this study, we characterized a molecule not yet described that we have named LIMLE and that we have identified as being over-expressed in the blood and graft of recipients developing rejection. We showed that LIMLE is expressed by myeloid cells, notably by DCs, following stimulation with pro-inflammatory reagents. In contrast, LIMLE expression is not increased and even tends to decreased by the immuno-regulatory cytokine IL-10. Similarly, LIMLE gene has been reported to be inhibited by the anti-inflammatory agent arbutin [45], [46]. This regulation of expression could explain the accumulation of LIMLE in recipients developing rejection versus tolerance. Indeed, rejection is characterized by the expression of pro-inflammatory cytokines and by the activation of myeloid cells that infiltrate the graft, uptake donor antigen and migrate to the secondary lymphoid organs to stimulate T cells [47]. In contrast, tolerance is associated with a strong inhibition of inflammation and cell activation and by the over-expression of immuno-regulatory factors that could repress LIMLE expression [8], [48], [49], [50].

We observed co-localization of LIMLE with actin at some membrane protrusions. Moreover, LIMLE is highly expressed in activated myeloid cells, ciliated cells [30], and in the testis, trachea and lung which are rich in highly ciliated cells. These data suggest a possible involvement of LIMLE in dynamic cell extensions. LIMLE and its neighboring genes IL-16 and Mesdc-1, that exhibit shared synteny between species, may form a complex of molecules sharing a functional relationship and the common feature of being associated with the cytoskeletal network.

Interestingly, LIMLE predictive structure study indicated a similarity of structure with trefoil proteins, some of which interact with actin. Among them are the actin filament (F-actin) bundling proteins Fascins [51] that exhibit the same expression profile as LIMLE, with a high expression in activated DCs (Fascin-1) [6] and a high expression in testis (Fascin-3) [52]. Fascin-1 is involved in the formation of filopodia, dendrites and invadopodia [53], [54], [55] that play critical roles in maturation-associated DC functions such as migration, cytokine production and interaction/activation of T cells [55], [56], [57], [58]. Interestingly, Fascin-3, like LIMLE, is highly expressed in testis at the spermatid stage ([52] and GEO profiles, data not shown). Indeed, during spermiogenesis, round spermatids are remodeled into the fusiform shape of mature spermatozoa. For that, significant morphological changes occur and are correlated with a reorganization of microfilaments and microtubules in the head and tail regions of elongating spermatids by the expression of specialized actin- and tubulin-associated proteins [52]. LIMLE, like Fascin, may function in the microfilament rearrangements that accompany fertilization. The role of these molecules sharing the common feature of being associated with the cytoskeletal network is important to decipher. Indeed, Fascin has received a lot of attention because multiple clinical studies have implicated its expression in cancer progression and metastasis [53]. We observed LIMLE protein also in the nucleus and identified a NES signal that allows molecule exchange between the cytoplasm and nucleus through a nuclear pore complex. Similarly to actin, which contains two NES [59], LIMLE may shuttle between the cytoplasm and nucleus to play a role in both compartments. Indeed, in addition to playing a fundamental role in the cytoplasm, actin has been described to bind RNA polymerase and to promote chromatin remodeling [60], [61], [62], [63].

We observed no role for LIMLE in the up-regulation of co-stimulatory molecules or in cytokine expression in DCs in response to TLR-L or in MLR. However, inhibition of LIMLE in DCs reduces the proliferation and IFNγ secretion of responding effector T cells. Therefore, LIMLE is not involved in DC maturation but rather plays a role in their ability to stimulate T cells. LIMLE, which is strongly up-regulated in DCs following inflammatory maturation, could be required for immune synapse formation with T cells to efficiently prime T cells. Indeed, recent studies have assigned an active role of DC actin cytoskeleton during immune synapse formation between T cells and DCs to allow efficient T cell activation [64]. We did not observe an effect of LIMLE expression in the expansion of regulatory T cells. Moreover, the immuno-regulatory cytokine IL-10 tends to decrease the expression of LIMLE suggesting that LIMLE inhibition could prevent expansion of effector T cells while sparing regulatory T cells. Therefore, a different expression of LIMLE in mature versus immature DCs may promote differently the formation and the stability of the immune synapse to generate either classical effector or regulatory T cells [65], [66]. For example, it has been shown that the protein kinase C by its different localization in the immune synapse between effector and regulatory T cell offers therapeutic implications to block undesirable inflammatory responses [67]. Indeed, inhibition, in effector T cells will reduce effector function, and in regulatory T cells will increase suppressor function. LIMLE by acting on DC-T cell interaction may also modulate the fate of effector T cells versus regulatory T cells and therefore the nature of the adaptive immune response. Indeed, it has been shown that BMDC deficient in the Wiskott-Aldrich Syndrome Protein (WASP), a regulator of the actin cytoskeleton reorganization, present no alteration of maturation or antigen endocytosis and processing but have a reduced ability to stimulate specific T cells due to an alteration in the immune synapse structure [58]. Interestingly, it has been shown that formation of the immunological synapse bears striking similarities to cilia formation and cytokinesis [68]. All share the unusual property of forming around the site of centrosomal docking at the plasma membrane. The synapse is a focal point for both exocytosis and endocytosis, both of which are triggered by localized cell signaling at the plasma membrane. Signaling triggers massive reorganization of both the actin and microtubule cytoskeletons, with the centrosome moving right up to plasma membrane allowing the synapse formation. LIMLE may play a role in this focal point important for cilia, cytokinesis and synapse formation. It will be interesting to further investigate the role of LIMLE in DC/T cell immune synapse formation. We did not observe an effect of LIMLE inhibition on DC migration in chemotaxis experiments (data not shown). This does not exclude a role for LIMLE in cytoskeleton rearrangement that is important for motility or mobility.

To conclude, we describe in this study a new molecule strongly expressed by inflammatory activated DC and important for efficient T cell priming. LIMLE could be involved in the dynamic function of the specialized structure of the cytoskeleton, important for cell adhesion, migration, motility or immune synapse formation in these cells. In the future, LIMLE may represent a new therapeutic target to regulate DC activity and immune responses.

## Supporting Information

Figure S1 **A**) Viability of rat LPS-stimulated BMDC (transfected 48 hours with control or two LIMLE specific RNAi). Data were analyzed by DAPI exclusion by flow cytometry and are expressed in percentage of live cells. Data are shown as the mean +/− SEM of 3 independent experiments. **B**) Dot plot gate used and representative histograms of MHC class I, MHC class II, CD80 and CD86 expression by rat unstimulated or LPS-stimulated BMDCs transfected 48 hours with control or two LIMLE specific RNAi. Analysis was performed by flow cytometry. The same results were obtained in five independent experiments. **C**) Quantification of IL-6, IL-12, IL-10 cytokine production by rat LPS-stimulated BMDC (transfected for 48 hours with control or two LIMLE specific RNAi). Data were quantified by ELISA (BD Biosciences) and are shown as the mean ± SEM of n = 3 samples pooled from three independent experiments.
(TIF)

Figure S2 **A**) Viability of mouse LPS-stimulated BMDC (transfected 48 hours with control or two LIMLE specific RNAi). Data were analyzed by DAPI exclusion by flow cytometry and are expressed in percentage of live cells. Data are shown as the mean ± SEM of 7 independent experiments. **B**) Dot plot gates used and representative histograms of MHC class I, MHC class II, CD80 and CD86 expression by mouse unstimulated or LPS-stimulated BMDCs (gated on CD11c^+^) transfected with control RNAi or two LIMLE specific RNAi. Analysis was performed by flow cytometry. Same results were obtained in five independent experiments.
(TIF)

Figure S3 **A**) Gate strategy, Representative flow cytometry analysis gates used to quantify the proliferation of OT-I T cells stimulated in vitro with mouse transfected BMDC (shown in figure 2F). Arrows show next gate. **B**) Dose-effect of proliferation of OT-I CD8+ T cell stimulated (for 4 days) with OVA257–264 peptide (0.001-10 ng/ml) or OVA protein (0-1000 μg/ml) loaded mouse LPS-stimulated BMDCs (transfected for 24 h with control or two LIMLE specific RNAi). Data are shown as the mean ± SEM n = 2 samples from one experiment representative of four independent experiments. Data were expressed as % of proliferation (CFSE dilution).
(TIF)
